# A machine learning algorithm supports ultrasound-naïve novices in the acquisition of diagnostic echocardiography loops and provides accurate estimation of LVEF

**DOI:** 10.1007/s10554-020-02046-6

**Published:** 2020-10-08

**Authors:** Matthias Schneider, Philipp Bartko, Welf Geller, Varius Dannenberg, Andreas König, Christina Binder, Georg Goliasch, Christian Hengstenberg, Thomas Binder

**Affiliations:** Department of Internal Medicine II, Medical University of Vienna, Waehringer Guertel 18-20, 1090 Vienna, Austria

**Keywords:** Left ventricular function, LVEF, Echocardiography, Machine learning, Artificial intelligence

## Abstract

Left ventricular ejection fraction (LVEF) is the most important parameter in the assessment of cardiac function. A machine-learning algorithm was trained to guide ultrasound-novices to acquire diagnostic echocardiography images. The artificial intelligence (AI) algorithm then estimates LVEF from the captured apical-4-chamber (AP4), apical-2-chamber (AP2), and parasternal-long-axis (PLAX) loops. We sought to test this algorithm by having first-year medical students without previous ultrasound knowledge scan real patients. Nineteen echo-naïve first-year medical students were trained in the basics of echocardiography by a 2.5 h online video tutorial. Each student then scanned three patients with the help of the AI. Image quality was graded according to the American College of Emergency Physicians scale. If rated as diagnostic quality, the AI calculated LVEF from the acquired loops (monoplane and also a “best-LVEF” considering all views acquired in the particular patient). These LVEF calculations were compared to images of the same patients captured and read by three experts (ground-truth LVEF [GT-EF]). The novices acquired diagnostic-quality images in 33/57 (58%), 49/57 (86%), and 39/57 (68%) patients in the PLAX, AP4, and AP2, respectively. At least one of the three views was obtained in 91% of the attempts. We found an excellent agreement between the machine’s LVEF calculations from images acquired by the novices with the GT-EF (bias of 3.5% ± 5.6 and r = 0.92, p < 0.001 in the “best-LVEF” algorithm). This pilot study shows first evidence that a machine-learning algorithm can guide ultrasound-novices to acquire diagnostic echo loops and provide an automated LVEF calculation that is in agreement with a human expert.

## Background

Left ventricular ejection fraction (LVEF) is by far the most important parameter in the assessment of cardiac function. Echocardiographic calculations of LVEF play an important role in diagnosis and clinical decision-making. This is especially true in cardiac device therapy, management of valvular heart disease, oncology patients, and in the diagnosis and treatment of heart failure [1–3]. At the same time, calculation of LVEF is strongly dependent both on image quality and on the experience of the reader [4]. Thus calculation of LVEF by non-cardiologists and beginners is a significant limitation for the widespread application of this parameter [5].

**Fig. 1:**
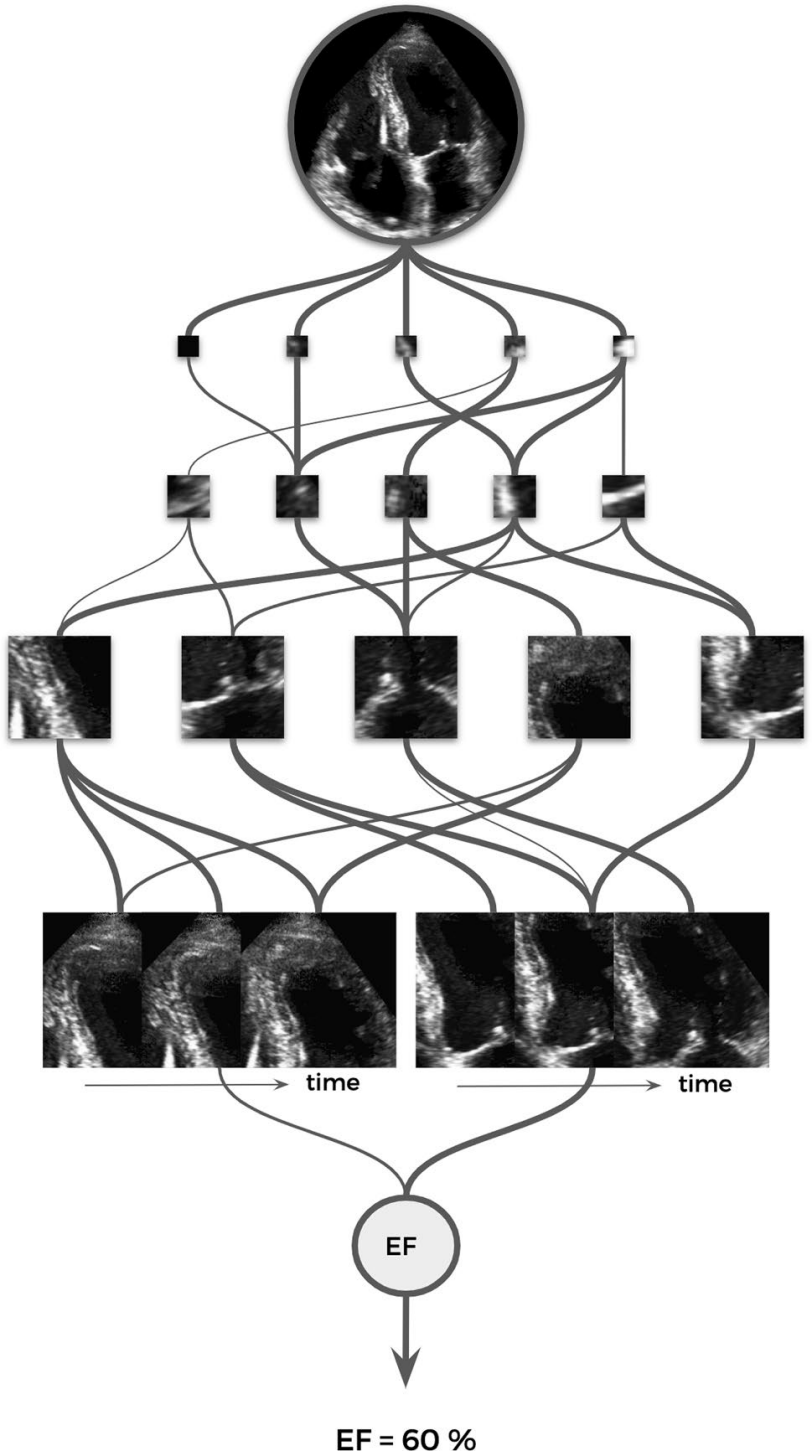
This schematic represents the multiple layers and nodes of a neural network with a sample echocardiography image being processed in order to produce an estimate of left ventricular ejection fraction

Quantitative evaluation of LVEF is performed by either human or automated computer-based measurements of end-diastolic and end-systolic volumes through the tracing of endocardial borders followed by model-based calculations. Recently, a new software was developed, implementing a fully machine-learning algorithm mimicking the human eye by estimating LVEF from the degree of ventricular expansion and contraction, myocardial thickening, and motion of the mitral annular plane (Fig. 1) [6]. This algorithm has meanwhile been developed further, also integrating the parasternal long axis view into global LVEF assessment.

**Fig. 2:**
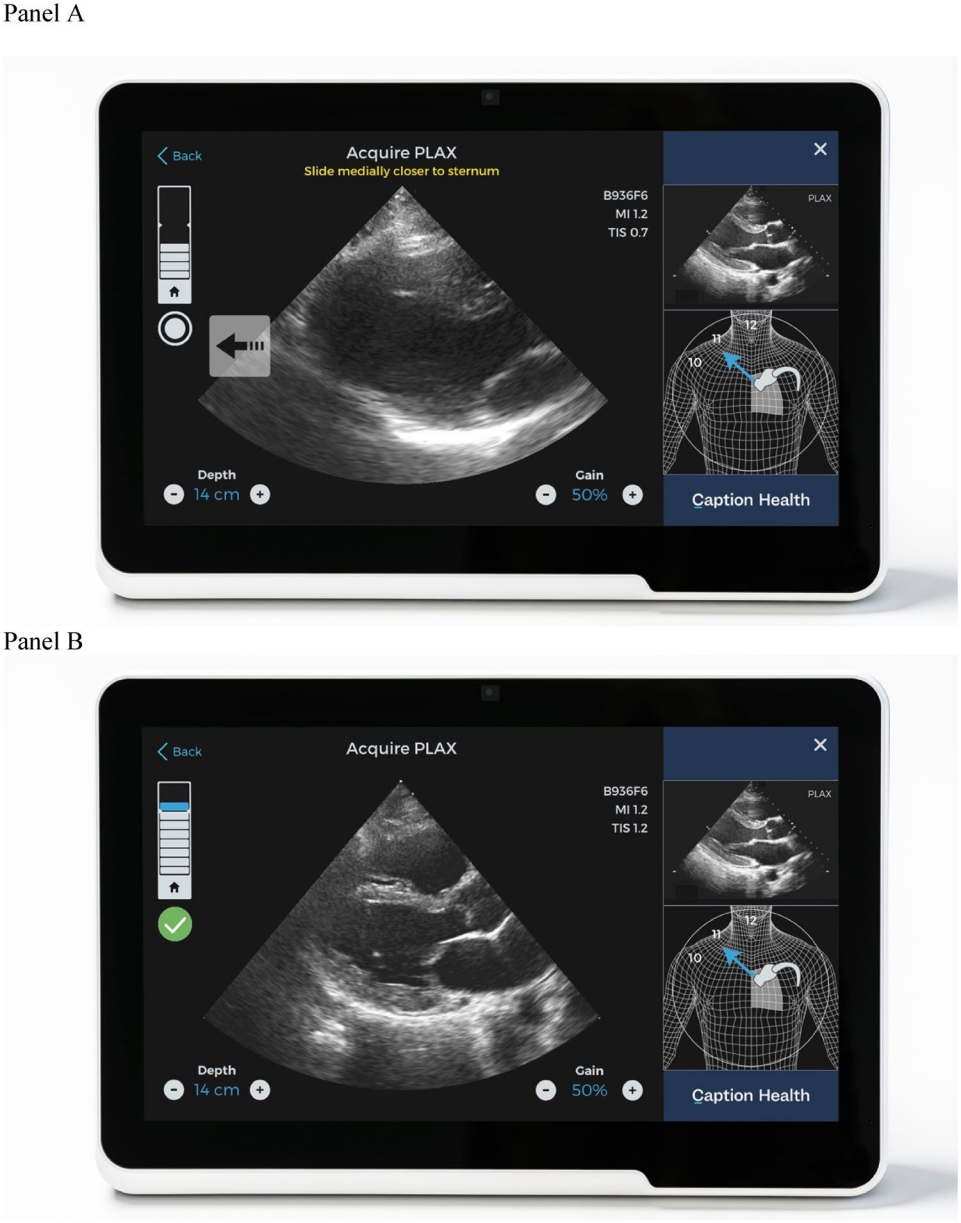
Panel **a** shows the guidance phase: The image is not optimal and the guidance message is shown (“slide medially closer to the sternum”). The quality meter shows that it has not reached the automated capture level. Panel **b** shows the screen when a good view has been obtained and automated capture has occurred. *PLAX* parasternal long axis view

Furthermore, another artificial intelligence (AI) algorithm was added, which was trained to guide the echocardiographer to acquire diagnostic images. Depending on the live-image, the machine advises the examiner to move, tilt, or rotate the transducer to reach the best image quality. While imaging, the machine automatically saves the best image acquired (Fig. 2).

A methodology, which allows even novices to acquire images that provide an accurate calculation of LVEF is revolutionary and could greatly impact the reach of echocardiography.

In our study we evaluated (1) if this new machine-learning algorithm is able to assist novice operators to acquire diagnostic image loops and (2) if the AI based algorithm using these images provides LVEF calculations comparable to those obtained by experts.

## Methods

All patients provided signed informed consent, and the study was approved by the local ethics committee (EK # 1833/2019). The study protocol conformed to the ethical guidelines of the Declaration of Helsinki.

### Performance testing

The design of this study consisted of four steps: (1) nineteen echo-naïve first-year medical students were briefly trained in the basics of echocardiography, (2) the trained students scanned three patients each with the help of the machine-learning algorithm (Caption Health Inc., Brisbane, CA, USA), for each patient they attempted to acquire three views (parasternal long axis view = PLAX, apical four-chamber view = AP4, apical two-chamber view = AP2), (3) the same patients were additionally scanned by one expert cardiologist (MS), the acquired loops were read by three blinded expert cardiologists (MS, TB, PB), the average calculated LVEF was taken as the ground-truth LVEF (GT-EF), (4) the 171 loops which were scanned by the students were evaluated by the machine-learning algorithm. If image quality was sufficient, LVEF was calculated analyzing PLAX only, AP4 only, AP2 only, and all acquired views together (“best-EF”). The machine’s results were compared to the experts’ GT-EF.

### Patients

Fourteen consecutive patients were included in this study. The patients were inpatients on the general cardiology ward at the time of the study. The study team had no prior knowledge on image quality or LVEF of these patients. Only patients in sinus rhythm were included.

### Novices and training

**Fig. 3:**
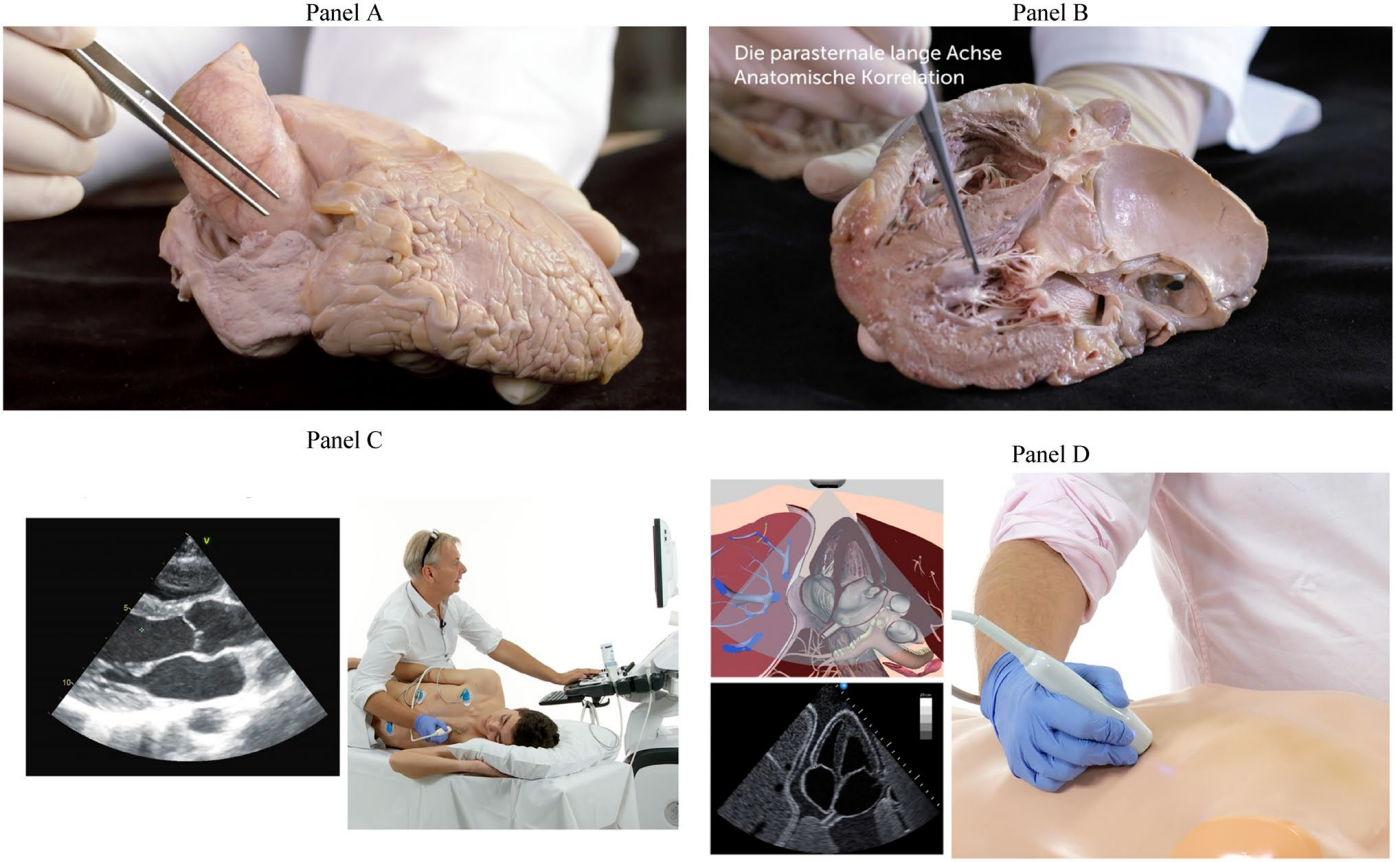
The videos include 2.5 h of video lectures, which explain cardiac anatomy in autopsy specimens (Panel **a** and **b**), recorded video demonstrations showing the screen and the transducer position (Panel **c**), and echo simulators (Panel **d**)

Nineteen echo-naïve first-semester medical students (first month) received a brief dedicated echo training. To provide basic knowledge we trained the students with a 2.5-h online echo tutorial (produced in cooperation between the Medical University of Vienna and www.123sonography.com), teaching the basics of cardiac anatomy, such as the position of the heart in the chest, morphology, and topography of the heart chambers and valves using human anatomic specimens, as well as video demonstrations of the echo cut-planes using a split-screen format that shows the transducer position and the images (Fig. 3). Ten of the students (trained novices) received an additional 2-h hands-on training with the machine-learning software, which allowed each student to image for 30 min. The scanning for this study was performed 2 and 3 days after the online and hands-on training, respectively.

### Scanning by the novices

Scanning was performed on a Terason uSmart 3200t (Burlington, MA, USA) tablet ultrasound system. The 19 novices scanned three patients each. Each student was asked to acquire the three views PLAX, AP4, and AP2 in each patient, supported by the machine-learning algorithm. The algorithm steers the user to the correct image through simple commands such as “tilt down”, “rotate clockwise”, etc. It denotes quality of the live image by displaying a visual score on a bar (“quality meter”), and automatically captures the loop with highest quality (Fig. 2). A total of 171 image loops were recorded by the students. Time to acquire each loop was recorded by the system.

### Reference loops by the expert

All patients were additionally scanned with a GE S70 (General Electric Healthcare, Chicago, IL, USA) machine to acquire reference images (PLAX, AP4, AP2). These images were read by three expert cardiologists (MS, TB, PB) who were blinded to the results provided by the artificial intelligence. Biplane Simpson LVEF was calculated for each patient by measuring end-systolic and end-diastolic volumes. The three LVEF values were averaged; the average was used as reference value for LVEF in this study (ground truth EF [GT-EF]). The expert additionally scanned all patients with the Terason machine to allow AI calculations on the expert loops as well.

### Image quality score

The Image Quality Score (IQS) technology provides an algorithmic determination of the diagnostic quality of an ultrasound image. The algorithm works in two stages. In the first stage, a Deep Convolutional Neural Network performs an inference over a given video clip to produce video clip embeddings. A second stage algorithm then takes these embeddings and produces an estimate of Image Quality Score (IQS) for that clip. The first stage algorithm was trained on a very large and diversified dataset of more than 400,000 clips from 12,000 unique patients (totaling approximately 17.7 million frames). The dataset was balanced over patient sex, age, body mass index, and ultrasound equipment systems. The second stage algorithm was trained on over 1000 unique clips for each view of interest (namely AP2, AP4 and PLAX) to regress the ground truth IQS scores collected from experienced echocardiographers. The second stage algorithm predicts an image quality score from 1 to 5 (i.e. a floating point number). The scale has been developed by the American College of Emergency Physicians [7].

To validate the algorithm, five expert registered cardiac sonographers reviewed a dataset of over 2400 pairs of same-view clips from patient studies that included AP4, AP2, and PLAX views. The sonographers scored each pair by telling if the left clip was of higher quality, the right clip was of higher quality, or if both clips were equally good or bad. The difference in image quality scores produced by the algorithm was computed for each pair and the resulting value was then mapped onto either − 1 (which meant that the left clip was deemed of higher quality by the algorithm), 0 (if both clips were considered of similar quality by the algorithm), or + 1 (which meant that the right clip was deemed of higher quality by the algorithm). The test evaluated the ability of the algorithm to estimate image quality differences with at least 80% of the performance of expert sonographers, this being considered acceptable performance. This target was met for the three views (PLAX 90.4%, AP4 95.0%, AP2 83.2%.)

In this study, IQS levels were used as a threshold to select clips considered to have sufficient quality for the algorithm to accurately perform the automated LVEF measurement. Thresholds for IQS were 3.1 for PLAX, 2.5 for AP4, and 2.7 for AP2.

### Artificial intelligence: guidance and LVEF calculation

The AI algorithm allows calculation of LVEF for each view (PLAX, AP4, AP2) as well as an aggregate LVEF based on all acquired views (“best-EF”). The “best-EF” can only be computed if more than one view provides diagnostic quality. At the time of the study, the LVEF tool was not yet implemented into the acquisition software. Therefore, LVEF calculation was not performed simultaneously but later via post-processing.

The algorithms for guidance and LVEF calculation were implemented in Python and trained using Keras (https://keras.io/) with a Tensorflow (https://www.tensorflow.org/) backend to train and deploy the neural networks. Details regarding training the machine have been published previously [6].

### Statistical analysis

Continuous variables are given as mean ± standard deviation (SD). Biases between the machine’s reads from the images acquired by novices and the expert and the expert-provided reference standard were tested using 2-tailed paired Student’s t-tests. Bland–Altman analysis was calculated and is provided as plot figure. Pearson correlation coefficients were calculated. Root mean square deviation (RMSD) was calculated as a measure of differences between the GT-EF and the three views PLAX, AP4, AP2, as well as the best-EF as estimated by the AI. A *p* value ≤ 0.05 was considered statistically significant. SPSS Version 24 (IBM SPSS, Armonk, NY, USA) was used for all analyses.

## Results

### Patients

Fourteen consecutive patients (one female, median age 55 years, interquartile range [IQR] 39–64, mean LVEF 58.5% ± 13, range 26–70) were included in this study. The patients were inpatients at the time of the study due to a variety of cardiac diseases and interventions, such as coronary artery disease, pulmonary vein isolation due to paroxysmal atrial fibrillation (all in sinus rhythm at the time of image acquisition), and PFO closure. The cohort also included three healthy volunteers. Each of the patients was scanned by at least three novices.

### Differences between the two groups of trained novices

There were no differences between those novices who received additional hands-on training (trained novices) when compared to those who received only the online-training (data not shown). In the following, the results of the 19 students will be presented together.

### Image quality

The software assessed the quality of the acquired images. If the quality score reached the diagnostic level, the machine performed LVEF calculations. The expert acquired diagnostic quality in 12/14 (86%), 14/14 (100%), and 14/14 (100%) patients in the PLAX, AP4, and AP2, respectively. The 19 novices each scanned three patients, with a total of 57 examinations. At least one of the three views PLAX, AP4, or AP2 was obtained by the novices in 91% of the attempts. One diagnostic image was acquired in 8/57 (14%), two of the three in 19/57 (33%), and all three in 25/57 (44%) of the scans (Table 1).

**Table 1:** Successful capture of diagnostic image quality loops including the scanning time for each view

	Expert	Trained novice	Novice
Number of successful captures of the view	N = 1 examiner 14 scans	N = 10 30 scans	N = 9 27 scans
PLAX, n (%)	12 (85.7)	17 (56.7)	16 (59.3)
AP4, n (%)	14 (100)	25 (83.3)	24 (88.9)
AP2, n (%)	14 (100)	21 (70)	18 (66.7)
Number of successful captures per patient			
0, n (%)	0 (0)	3 (10)	2 (7.4)
1, n (%)	0 (0)	4 (13.3)	4 (14.8)
2, n (%)	2 (14.3)	10 (33.3)	9 (33.3)
3, n (%)	12 (85.7)	13 (43.3)	12 (44.4)
Time to capture the view			
PLAX, s (± SD)	48 (± 38)	191 (± 113)	186 (± 143)
AP4 s (± SD)	20 (± 13)	230 (± 174)	181 (± 132)
AP2 s (± SD)	30 (± 19)	141 (± 89)	140 (± 89)

The percentage numbers are referring to the number of scans performed in each group. The examiner scanned all 14 patients, the novices each scanned three patients

*PLAX* parasternal long axis view, *AP4* apical four chamber view, *AP2* apical two chamber view, *s* seconds, *SD* standard deviation

In total, the novices acquired diagnostic quality in 33/57 (58%), 49/57 (86%), and 39/57 (68%) acquisitions in the PLAX, AP4, and AP2, respectively.

### Time for image acquisition

Time to acquire each loop was compared. For the novices, time to acquire PLAX, AP4, and AP2 was 189 (± 127), 207 (± 156), and 141 (± 88) seconds, and for the expert 48 (± 38), 20 (± 13), and 30 (± 19) seconds (difference between the groups p < 0.001), (Table 1).

### LVEF results: bias and correlation

**Fig. 4:**
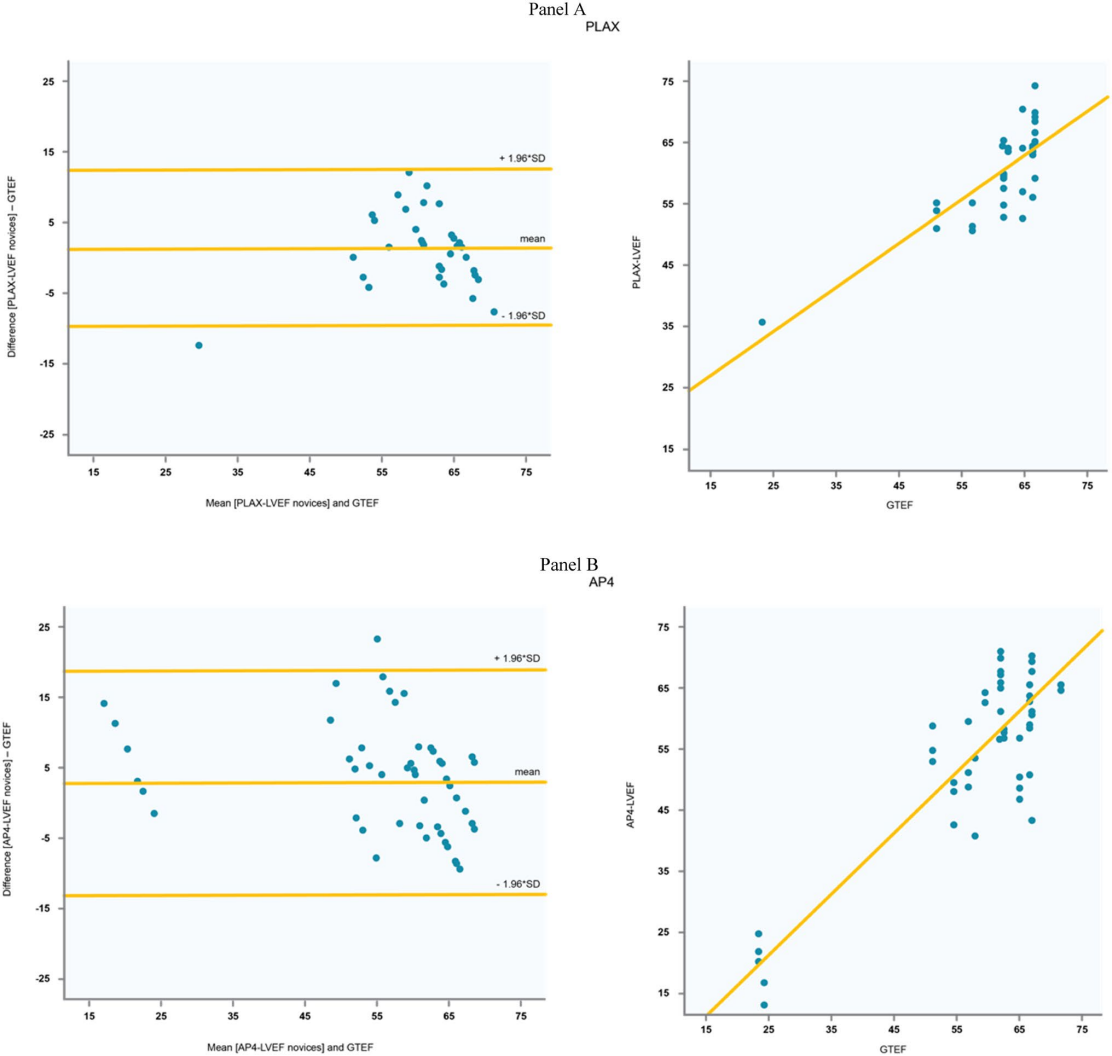

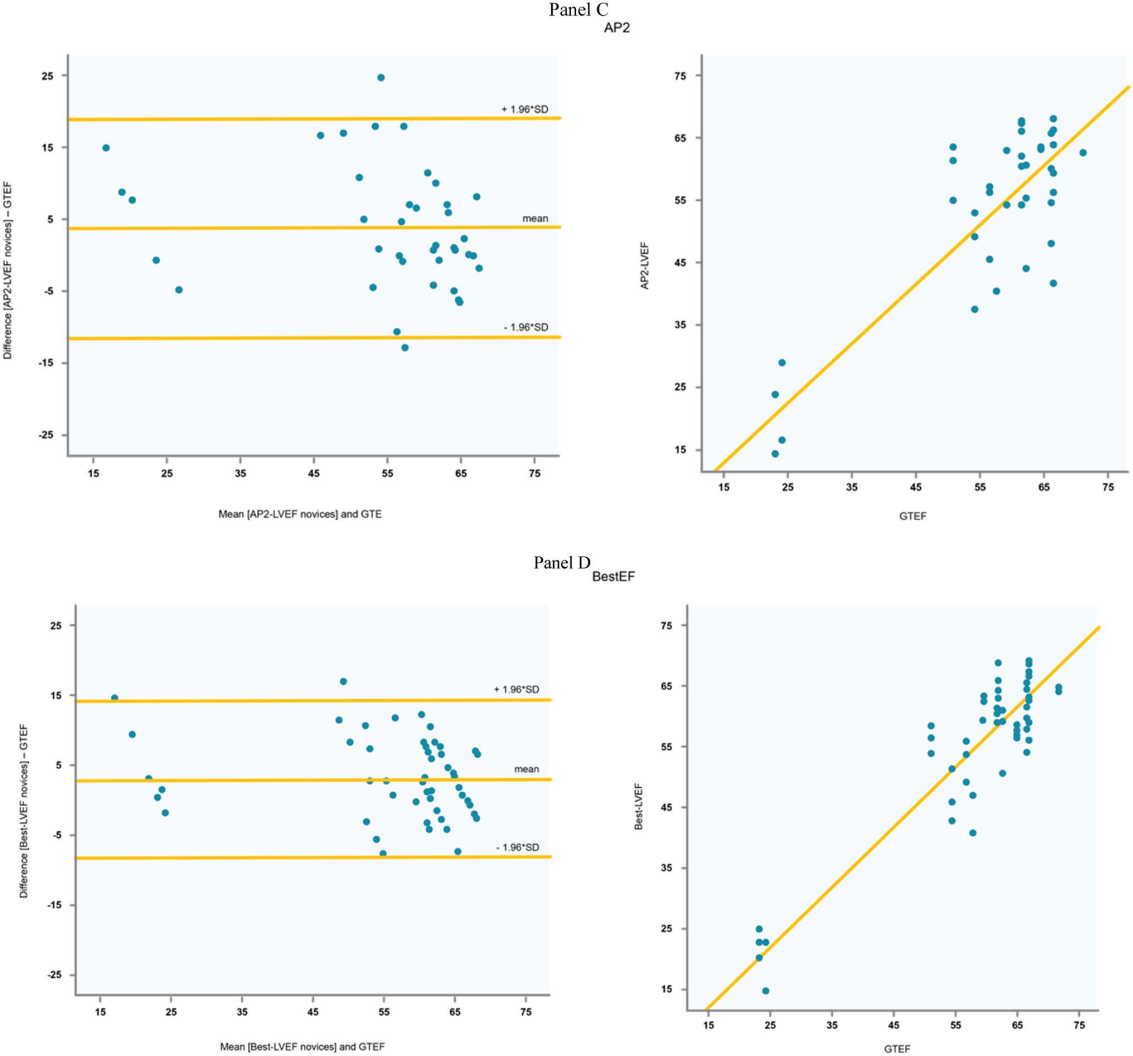
Bland Altman plots and correlation diagrams depicting the interrelation of ground-truth left ventricular ejection fraction (GTEF) with the artificial intelligence derived calculations. Panel **a** Parasternal long axis view (PLAX) and GTEF. Panel **b** Apical 4-chamber view (AP4) and GTEF. Panel **c** Apical 2-chamber view (AP2) and GTEF. Panel **d** Best-LVEF = Multiplane ejection fraction (mean of all available view if more than one view could be obtained) and GTEF

Automated measurements of loops acquired by the novices were in excellent agreement with the experts’ GT-EF. This was reflected by a bias of 1.35% (± 5.63), with a correlation coefficient of r = 0.79 (p < 0.001) in the PLAX views, a bias of 3.05% (± 8.1) and r = 0.87 (p < 0.001) in the AP4 views, a bias of 3.94% (± 7.8) and r = 0.84 (p < 0.001) in the AP2 views, and a bias of 3.02 (± 5.7) and r = 0.92 (p < 0.001) in the “best-LVEF” algorithm (Fig. 4). For PLAX, AP4, AP2, and “best-EF”, RMSD was 5.3%, 8.4%, 9.2%, and 6.5% for the novices and 6.7%, 9.1%, 6.8%, and 6.2% for the expert.

## Discussion

In this study we could show that minimal online training in combination with a machine-learning algorithm can guide echo-novices to acquire diagnostic echo studies. An artificial intelligence algorithm can produce an LVEF estimation from these captured images that is in agreement with an expert echocardiography specialist. This finding has the potential to change the clinical practice of echocardiography.

Initially a highly specialized diagnostic tool performed only by cardiologists, echocardiography has already spread to other medical specialties. With a drop in cost, size, and availability of ultrasound scanners, echocardiography is now being performed also in primary and emergency care settings. It has been speculated that in the near future hand-held-ultrasound will replace the stethoscope [8]. However, this raises concerns that the level of training of health care professionals performing ultrasound is not sufficient to yield accurate and reliable results. It is of particular concern that inaccurate quantification of LVEF may lead to false decisions, which could potentially harm patients and increase health care cost.

To truly bring echocardiography to the forefront of medicine it is therefore important to reduce training time, improve the learning curve, and to use standardized analyzing tools, which make the technique less operator dependent. Regarding LVEF, it is important to provide accurate calculations with high reliability.

Artificial intelligence and machine-learning promise to provide methodologies, which aid in image optimization, analysis, and interpretation [9]. In this study, we tested a new machine-learning algorithm which applies artificial intelligence both in helping echo-naïve novices to acquire loops of diagnostic quality and in assisting the observer by estimating LVEF. Opposed to conventional methods which use endocardial border tracing, the algorithm applies “pattern recognition” to mimic the human approach of “eyeballing”.

### Artificial intelligence assisting echo-naïve students to acquire diagnostic images

Untrained users often struggle with the acquisition of diagnostic echo images. Currently, the method requires highly trained specialists to perform and interpret the study. Multiple strategies have been proposed to standardize optimal image acquisition in echo. This includes position localizers, 3D echocardiography, and mechanical steering arms. However, these methodologies are complex, expensive, and cannot be used bedside and in emergency settings.

The new system tested in this study applies an inbuilt machine-learning algorithm that guides the operator to the correct transducer position, detects when the optimal image orientation and image quality is achieved, and automatically saves the best image, thereby guaranteeing that the best loop is acquired. Once stored, the algorithm provides an estimation of LVEF.

In our study, we recruited 19 random first-semester medical students in the first month of their studies on a first-come first-serve basis. None of them had previous knowledge of ultrasound. We trained the novices with a 2.5 h online teaching tool in anatomy of the heart and in basics of echocardiography. An additional 2-h hands-on training was performed with 10 of the students.

Time to acquire diagnostic images was—as expected—significantly longer for the novices when compared to the expert. It took the novices between 2.5 and 4 min to acquire each individual loop, the expert needed a mean of 32 s. However, it must be stressed that the novices have never previously imaged a patient. Diagnostic image quality (at least one of the three views) could be obtained in most patients (91%). This demonstrates that the system allows even novices with minimal training to perform an echo study of sufficient quality for LVEF assessment, though naturally not with the same results as an expert.

This finding can have direct implications on scenarios such as the current COVID-19 pandemic. Depending on the hospital setting, the number of imaging specialists is limited. Ideally, bed-side ultrasound is performed in contagious patients to reduce the number of exposed health care professionals. The guidance system allows a one-stop-shop approach where one person not necessarily trained in ultrasound performs multiple tasks, including acquisition of echo loops, which can be read by an expert in a remote room.

### Artificial intelligence to estimate left ventricular ejection fraction

When performed by humans, echocardiography and its measurements are subjective and greatly influenced by the level of expertise [5, 10]. Since its first description there have been concerns about the reproducibility of LVEF due to significant intra-observer, inter-observer, and inter-institutional variability of measurements [11–13]. Standardized training of novices can reduce this bias [14, 15].

LVEF itself has many limitations, especially in patients with moderate or poor image quality where endocardial tracing and volumetric measurements are difficult to perform [4]. In our study, the “best-LVEF” showed a bias of 3.02% ± 5.7 in loops acquired by first semester medical students. The results did not differ significantly from those performed by an expert cardiologist (2.3% ± 6.0).

It is important to stress, that the machine-learning measurements are not based on endocardial tracing and volume calculation but they mimic the human eye and give a true eyeballing estimate. Thus, it represents the cumulative knowledge and experience of a senior cardiologist. The machine will eyeball with the same algorithm in every single calculation, thereby allowing standardizing this highly subjective method. The ability to estimate LVEF not based on volumetric parameters also offers the possibility to derive LVEF in patients where optimal (non-foreshortened) views cannot be obtained.

Recently, other studies could show the feasibility of a deep learning algorithm estimating two-dimensional LVEF, as well as three-dimensional right ventricular ejection fraction [16, 17]. In addition, pacemaker leads can be detected, cavity volumes can be estimated, and age, sex, and weight can be predicted [18]. Other groups trained their algorithm to detect patients with hypertrophic cardiomyopathy, amyloid heart disease, pulmonary arterial hypertension, and heart failure with preserved ejection fraction [19, 20].

This study demonstrates that the machine-learning algorithm can also obtain LVEF calculations from a parasternal view using a PLAX. This opens the window to include many more views into the calculation of LVEF, circumventing the limitation of biplane and apical methods where not all myocardial segments are included. The “best-LVEF” algorithm includes all acquired images (at this time PLAX, AP4, AP2) into the assessment. This could ultimately lead to a true global LVEF with far more clinical relevance than a biplane Simpson LVEF calculated through volumetric assumptions from an area traced by a human being or calculated from a semiautomatic method (“Auto-EF”). Thus, neural network trained algorithms might revolutionize the application of the parameter LVEF in the future.

A unique finding of this study is the ability of the algorithm to allow estimation of global LVEF from PLAX only. The PLAX has traditionally been excluded as a view from which LVEF should be calculated. The main reason being that opposed to the apical long axis (which has the same image orientation) it does not display the apical segments [21]. At the same time, in daily clinical practice, every operator will already eyeball global function in PLAX when starting to evaluate a dataset of echo loops. Therefore, it comes without surprise that a machine learning algorithm trained on tens of thousands of echo studies can give an estimation of LVEF when only confronted with a PLAX. Patients e.g. with apical aneurysm or inferior scars will have overestimated LVEF by this method, at the same time values for the general population will be quite reliable. In general, the more regional abnormalities are present, the more views are needed to account for true global ejection fraction. Thus, the inclusion of PLAX in this algorithm might lead to better reliability of the parameter LVEF.

The combination of a loop acquisition system with a data analysis software has the potential to revolutionize the training and practice of echocardiography. Clearly, the system not only allows less experienced operators to perform diagnostic quality studies but is also helpful in the training process itself. Similar to a human instructor the algorithm guides the hand of the trainee and thereby assists in hand–eye coordination. Our study also suggests that trainees with and without initial hands-on instruction perform equally well. The guidance algorithm in itself serves as hands-on instruction.

By relying less on highly trained personnel, more patients can be brought to the exam. Concerns that unexperienced observers will make wrong estimations on ventricular function can be alleviated by the fact that machine-learning based calculations increase accuracy and standardize image quality. Nevertheless, it must be emphasized, that at least at this time the automated diagnosis only includes assessment of LVEF, other pathologies might be missed. Therefore, this technique does not replace a regular echo exam. As the automated capture technique produces optimal image quality, this would allow a second reading, though. This may be a target for a more advanced AI algorithm in the future.

The findings of this study are hypothesis generating: In the future, general internists e.g. working in dialysis or oncology, might perform screening studies which select those patients who need urgent TTE performed by cardiologists, and at the same have the opportunity to document stable LVEF in patients who would need but—in current clinical practice—do not receive several follow-up TTE studies (especially in oncology). This can lower the threshold to perform TTE, thereby improving the level of patient care. More data is needed to test these visions.

### Limitations

This study included only a small number of novices and patients. However, since each novice performed multiple views and scanned three patients each, we believe the data is robust enough to draw the outlined conclusions and formulate our visions.

Our data does not represent the full spectrum of different ejection fractions. Thus, it is not entirely clear how the system performs in the various categories of left ventricular dysfunction. Therefore, the results of this study are primarily hypothesis generating. They must be studied and reproduced in future studies with a wider spectrum of pathologies.

A success rate of 58% capturing the PLAX view with—at the same time—significantly higher success rates in the AP2 and AP4 views seems unusual. From our own teaching experience, the latter ones would be expected to be harder to obtain. One reason could be that two of the included patients had hard to obtain imaging conditions for the PLAX. This is also reflected by the fact that the expert failed to acquire diagnostic PLAX images in these two patients. Another reason might be the limited hands-on training. A reasonable but still modest amount of hands-on training might help improve the success rates. Future studies with larger numbers of patients and novices will help understand this matter better.

Only one female patient was included in the study. In males identification of chest landmarks are oftentimes easier than in females which might have led to a bias in analysis of the guidance system. This needs to be focused on in future prospective studies.

## Conclusion

This pilot study shows first evidence that a machine-learning algorithm can guide ultrasound-novices to acquire diagnostic echo loops and provide an automated LVEF calculation that is in agreement with a human expert. This has the potential to transfer the method and the parameter into other settings apart from a highly specialized echo lab.
